# Immunomodulation in Cutaneous T Cell Lymphoma

**DOI:** 10.3389/fonc.2019.01025

**Published:** 2019-10-09

**Authors:** Martina Ferranti, Giulia Tadiotto Cicogna, Irene Russo, Mauro Alaibac

**Affiliations:** Unit of Dermatology, University of Padua, Padua, Italy

**Keywords:** cutaneous T-cell lymphoma, immunosuppression, HIV, CD30 cutaneous T-cell lymphoma, mycosis fungoides

Primary cutaneous lymphomas (PCL), defined as lymphomas limited to the skin with no evidence of extracutaneous disease at the time of diagnosis, are a heterogeneous group of lymphoproliferative disorders. They encompass cutaneous T-cell lymphomas (CTCL) (65% of all cases), cutaneous B-cell lymphomas (CBCL) (25% of all cases) and other rarer variants (1, 2).

The most common CTCL subtype is mycosis fungoides (MF) which together with Sézary syndrome (SS) accounts for about 65% of all CTCLs (1). The neoplastic cells of CTCL usually express a CD3+CD4+ mature helper T-cell phenotype. CTCLs include also the group of primary cutaneous CD30+ lymphoproliferative disorders which represent at least 25% of all CTCLs (1–3). These conditions are generally characterized by expression of the CD30 molecule by more than 75% of neoplastic lymphocytes. In particular, the group of CD30+ lymphoproliferative disorders of the skin includes two subtypes: lymphomatoid papulosis (LyP) and primary anaplastic large cell lymphoma (PALCL) (4). However, these two entities differ significantly in several aspects. In contrast to LyP, PALCL may spread to extracutaneous sites in 10–20% of the patients, whereas the classical type of LyP is characterized clinically by recurrent self-healing lesions and there is no risk of spread to extracutaneous sites. The presence of clonality by TCR gene rearrangements is detected only in about 40% LyP cases in contrast to ALCL where it is observed in about 90% of cases (4). Moreover, in a study investigating the prevalence of PCLs in solid organ transplant recipients it has been demonstrated a significant increase of PALCL in this group of patients whereas only one case of LyP was reported (5). This is consistent with view that LyP is probably a reactive conditions whose onset is hampered by the immunosuppressive therapy, whereas ALCL is a true lymphoma and consequently its development is favored by the immunosuppressive regimen. On the other hand, there are data demonstrating an increased risk of MF in patients with LyP (6). Gene expression analysis in these disorders may help to shed light on this issue.

The role of viral agents in CTCLs is still debated, but it has not possible to date to convincingly establish a causal correlation between a viral infection and CTCL development (7). A recent study investigated CTCLs in HIV-infected and non-HIV-infected patients (8). Data were obtained from the Surveillance, Epidemiology, and End Results program, 1973–2013, of the U.S. National Cancer Institute and showed that HIV-infected patients with CTCL experienced significantly higher survival and a decreased risk of overall mortality than non-HIV-infected patients. Moreover, these results indicated that HIV infection was an independent protective factor. These epidemiological data suggest that a HIV-based therapeutic approach could be appropriate for CTCL. To this regard, previous studies generated a retroviral vector which specifically transfers genes into CD4+ cells by pseudotyping of murine leukemia virus (MLV) capsid particles with a variant of the HIV-1 envelope protein (9, 10). These vectors are suitable for gene therapy of CTCL, because they are able to deliver therapeutic genes exclusively to target cells. A subsequent study developed a xenograft mouse model to study CTCL and generated MLV/HIV-pseudotyped vectors encoding the herpes simplex virus thymidine kinase suicide gene (HSV-TK) (11). Vector particles were administered intratumorally into human CTCL xenografts in nude mice which then underwent systemic treatment with ganciclovir (GCV). Tumor development was significantly delayed in HSV-TK-transduced and GCV-treated tumors. These data demonstrate that the use of MLV/HIV-pseudotyped vectors could be an appropriate approach for the treatment of CTCLs.

Interferons (IFNs) are naturally occurring proteins released by host cells which act as a part of the innate immune response (12). Although IFNs were first identified for their capacity to induce resistance to virus, they also exert a wide range of immunomodulatory and antitumor effects (11). In particular IFN-alpha is an effective treatment for the two main subtypes of CTCL, MF and SS (13). To this regard, IFN alpha promotes the anti-tumor activity of NK cells and CD8+ T lymphocytes (12). However, the efficacy of IFN-alpha is limited by loss of response in a significant proportion of patients. An alternative or adjunctive strategy could be the utilization of the immune checkpoint inhibitors, such as anti-CTLA4 and anti-PD1/PDL1, which have become a new promising way of immunotherapy (14). CTLA4 and PD1 are two immunomodulatory receptors expressed on T cell and involved in the inhibition of immune system. The interaction between the receptors PD1 and CTLA4 and their ligands (PDL1 and CD80/CD86, respectively) induces a downregulation of T cell effector functions leading to the inhibition of the antitumor immune response (14). Thus, PD1 inhibitors (nivolumab and pembrolizumab) and CTLA4 inhibitors (ipilimumab) enhance anti-tumor immune response delaying tumor growth and facilitating tumor rejection. The relationship between immune checkpoint inhibitors and CTCL has not yet been clarified. A significant increase of PD1 expression in peripheral blood malignant T cells has been observed in SS, whereas data regarding overexpression of PD1 and CTLA4 in MF patients are conflicting (15). To this regard, the inhibition of PD1 and CTLA4 may have an important role in controlling the progression of some CTCL and could be investigated as a potential immunotherapy for SS and MF (16). Currently clinical trials over the use of pembrolizumab and nivolumab for the treatment of MF/SS are ongoing (17, 18). Promising results in the use of ipilimumab for the treatment of CTCL, specifically SS, have also been observed (19). Interestingly a case of complete regression of MF after ipilimumab therapy for advanced melanoma has been reported (20). On the other hand, a case of cutaneous CD56 + T cell lymphoma that developed during pembrolizumab treatment for metastatic melanoma has been described by Zheng et al. (21). Recently, the approval of mogamulizumab, a humanized defucosylated anti-CC chemokine receptor 4 (CCR4) monoclonal antibody, has expanded the landscape of drugs for the treatment of advanced CTCL (22). CCR4 is expressed in the vast majority of CTCLs, especially when peripheral blood involvement is present. This receptor plays an essential role in T-lymphocyte migration into the skin. In a recent study mogamulizumab significantly improved progression-free survival in advanced CTCL, especially in those with SS which is a subtype of CTCL characterized by peripheral blood involvement (23). Furthermore, a number of clinical trials are currently focusing on evaluating mogamulizumab with checkpoint inhibitors (NCT03309878, NCT02476123) as a means of improving antitumor immunity. For less advanced MF, topical resiquimod gel, a Toll-like receptor 7/8 agonist, has been used (24). This topical approach triggers innate immune responses that in turn support the induction of tumor-specific immunity. Lesions of MF treated with resiquimod gel significantly improved in 75% of patients and 30% of patients showed complete resolution of all treated lesions (25). Furthermore, in some patients resiquimod promoted distant response of untreated lesions indicating a systemic antitumor effect of the gel (25).

## Author Contributions

MA conceived the idea. MF, GT, IR, and MA contributed to the design of the article and to the writing of the manuscript.

### Conflict of Interest

The authors declare that the research was conducted in the absence of any commercial or financial relationships that could be construed as a potential conflict of interest.
